# Metabolic compatibility and the rarity of prokaryote endosymbioses

**DOI:** 10.1073/pnas.2206527120

**Published:** 2023-04-18

**Authors:** Eric Libby, Christopher P. Kempes, Jordan G. Okie

**Affiliations:** ^a^Department of Mathematics and Mathematical Statistics, Umeå University, Umeå 901 87, Sweden; ^b^Integrated Science Lab, Umeå University, Umeå 901 87, Sweden; ^c^The Santa Fe Institute, Santa Fe, NM 87501; ^d^School of Earth and Space Exploration, Arizona State University, Tempe, AZ 85287

**Keywords:** endosymbiosis, eukaryogenesis, metabolic model, prokaryote, evolution

## Abstract

The evolution of the mitochondria was a significant event that gave rise to the eukaryotic lineage and most large complex life. Central to the origins of the mitochondria was an endosymbiosis between prokaryotes. Yet, despite the potential benefits that can stem from a prokaryotic endosymbiosis, their modern occurrence is exceptionally rare. While many factors may contribute to their rarity, we lack methods for estimating the extent to which they constrain the appearance of a prokaryotic endosymbiosis. Here, we address this knowledge gap by examining the role of metabolic compatibility between a prokaryotic host and endosymbiont. We use genome-scale metabolic flux models from three different collections (AGORA, KBase, and CarveMe) to assess the viability, fitness, and evolvability of potential prokaryotic endosymbioses. We find that while more than half of host–endosymbiont pairings are metabolically viable, the resulting endosymbioses have reduced growth rates compared to their ancestral metabolisms and are unlikely to gain mutations to overcome these fitness differences. In spite of these challenges, we do find that they may be more robust in the face of environmental perturbations at least in comparison with the ancestral host metabolism lineages. Our results provide a critical set of null models and expectations for understanding the forces that shape the structure of prokaryotic life.

## Significance

Endosymbiosis is extremely rare in bacteria and archaea. Yet, one such endosymbiosis gave rise to eukaryotes and eventually complex multicellular life. While many factors may contribute to the rarity, we lack ways to estimate their influence. Here, we develop a quantitative approach to estimate the influence of one possible factor: metabolic compatibility, which is the ability of both host and endosymbiont to grow on the same resources. Using metabolic networks of existing prokaryotes, we find that more than half of possible endosymbioses would be metabolically viable; however, the resulting endosymbioses are less fit than their ancestors and unlikely to gain adaptations that overcome their fitness disadvantages. Our results provide null models that shed light on the diversity of prokaryotic life.

The evolution of mitochondria—from independent organism to intracellular organelle—is exceptional in terms of its physiological significance, ecological repercussions, and apparent evolutionary rarity (1–4). While prokaryotes and eukaryotes of similar cell size have roughly the same total metabolic rate (5, 6), the advantages conferred by the mitochondria, such as extra scale-free internal membrane area (7) along with distributed copies of the metabolic genes (8), may have facilitated the evolution and spread of large cells and complex multicellularity.

Moreover, after the establishment of the mitochondria, many branches of the eukaryotic lineage acquired other intracellular endosymbionts, including plastids (9), nitrogen-fixing bacteria (10), and methanogenic archaea (11). The impressive radiation of Eukaryota and the frequency by which the eukaryotic lineage has gained intracellular endosymbioses suggests that they confer evolutionary and ecological advantages (12, 13). Nevertheless, despite any possible advantages, the evolution of mitochondria is one of only a few reported cases of an endosymbiosis between prokaryotes (14–16), which is surprising given the opportunities afforded by their abundance and long evolutionary history. Why are there so few documented extant examples of prokaryote endosymbioses and why have they not achieved anything comparable to the remarkable ecological and evolutionary success of the eukaryotic lineage?

**Fig. 1.**
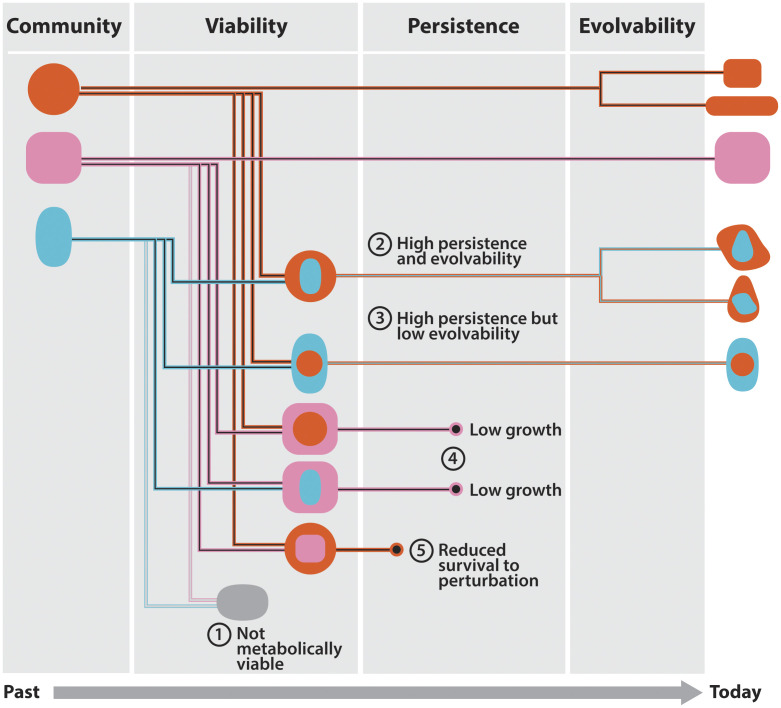
Stages in the evolutionary rise of endosymbioses. From its inception, a nascent endosymbiosis faces different barriers that challenge the success of its lineage. This schematic organizes these barriers into three broad stages corresponding to initial viability, persistence, and evolvability. Metabolic compatibility influences barriers in each of these stages. In the viability stage, both host and endosymbiont must be able to grow and reproduce such that the pair can produce offspring host–endosymbiont pairs. In the persistence stage, the population of endosymbioses must persist (avoid extinction) by surviving environmental perturbations and competing successfully with other species, including their ancestors. In the evolvability stage, the endosymbiosis must be able to access a sufficient number and caliber of beneficial mutations to foster adaptation to various environments. The successful navigation of evolutionary trajectories through these stages determines the abundance and diversity of endosymbioses in the biosphere.

Historically, these questions have been addressed by identifying possible barriers to the initial establishment of a prokaryotic endosymbiosis, such as the rarity of phagocytosis in modern prokaryotes (17) or issues with metabolic compatibility (18). Yet, the initial establishment is not the only stage at which a nascent endosymbiosis may encounter limitations. For example, a nascent endosymbiosis must be fit enough to compete with other organisms in the environment and it must also be able to adapt in order to spread into other environments and diversify (Fig. 1). At different stages, we may expect various ecological, physiological, or evolutionary constraints to be more dominant, but we currently lack quantitative approaches or null models to estimate the magnitude of these constraints or compare their influence. Thus, we have a fundamental knowledge gap concerning the forces that shape prokaryotic evolution, the origin of eukaryotes, and the distribution of endosymbioses in the biosphere. To address this knowledge gap, we need quantitative approaches that clarify the relative importance of the various barriers that limit the biosphere’s production of prokaryotic endosymbioses.

While in principle we could focus on any of the different barriers, one powerful approach is to focus on universal metabolic traits that apply across the biosphere’s ensemble of species and environments (19, 20). Since metabolism plays a central role in many ecological and evolutionary phenomena (21–24), a quantitative metabolic framework may offer key insights into the evolution of prokaryotic endosymbioses. Indeed, there are crucial metabolic considerations underlying each stage along the way from initiation to a persistent, evolvable endosymbiosis. For example, the initial viability of an endosymbiont (scenario (1) in Fig. 1) depends on whether it can access all of its required molecular compounds from within the host cell, which is governed by the spatial structure of their coupled metabolisms. The persistence of a host–endosymbiont pair, i.e., its ability to avoid extinction for an extended period of time, is determined by its fitness in different scenarios of survival and growth competition, which depends on whether the two metabolisms compete for resources or are able to synergize their metabolic pathways. Finally, the evolvability of an endosymbiosis is shaped by the degree to which the coupled host–endosymbiont metabolisms can harness the effects of mutations (scenarios 2 to 5 in Fig. 1). In general, it may be difficult to predict precisely the viability, persistence, and evolvability of a putative prokaryotic endosymbiosis because they depend on many idiosyncratic factors, such as the physical and ecological environment. However, we can draw upon the burgeoning wealth of genomic data and metabolic network models to elucidate the relative extent to which the metabolic underpinnings of viability, persistence, and evolvability hinder the establishment and radiation of prokaryote endosymbioses across the biosphere.

An important tool in quantifying the ecoevolutionary role of metabolism is the genome-scale metabolic model. Such models use the genomes of an organism to infer its metabolic repertoire and predict its growth in different environments. Genome-scale metabolic models are now available for thousands of species and have been successfully applied to a variety of bioengineering, ecological, and evolutionary problems (25, 26). Of particular relevance, they have also been used to accurately predict fitness and adaptive evolution in prokaryotic communities (27–31). In addition to modeling systems that can be experimentally validated, they have also been used to address questions that extend beyond our knowledge of and application to extant life. For example, metabolic models have been used to propose possibilities for early metabolism on Earth (32) and identify what metabolisms are likely to exist in distinct planetary conditions (33). Such generality and flexibility are ideal for our interest in exploring the challenges of an event that is possible but rarely seen.

Here, we harness the abundance and flexibility of genome-scale metabolic models to consider putative endosymbioses between random pairs of prokaryotes. By using different collections of metabolic models along with a large number of genomes, we are able to quantitatively study general features of metabolic compatibility in nascent endosymbioses and estimate viability, persistence, and evolvability in several combined outcomes as outlined in Fig. 1. In this analysis, we assess the extent to which metabolic compatibility acts as a barrier, independent of other considerations such as whether endosymbioses form through phagocytosis or via other ecoevolutionary processes (34). Ultimately, we find that the dominant metabolic barriers to a prokaryotic endosymbiosis relate not to its initial metabolic viability but its persistence and evolvability.

## Results

To assess metabolic compatibility in prokaryotic endosymbioses, we need a broad set of genome-scale metabolic networks (*Methods: Genome-Scale Metabolic Model Curation*). Thus, we obtained metabolic flux models for prokaryotes from three of the largest collections: AGORA (818 models), KBase (1,637 models), and CarveMe (5,587 models). A common feature across collections is that models organize reactions and compounds into compartments; all models have compartments for the cytoplasm and extracellular environment, but CarveMe models are the only ones with an additional compartment for the periplasm. The collections vary in what species, reactions, and compounds they include, as well as their description of how reactions and compounds behave across compartments. Since the collections also differ in their standards for metabolic model creation and formats, even if the same species appears in multiple collections, the metabolic models are not directly translatable. We manage this variation by keeping models from different collections distinct and performing analyses independently on each collection. This reduces the risk of introducing errors and provides an opportunity to assess the extent to which our results depend on metabolic model formats.

**Fig. 2.**
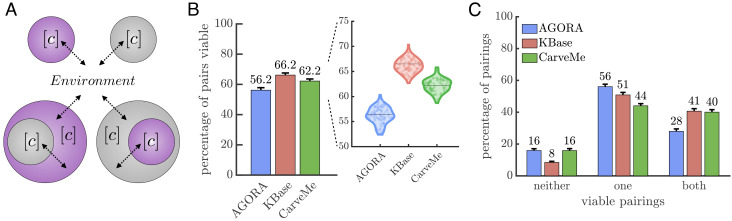
Viability of prokaryotic endosymbioses. (*A*) A schematic shows the nested compartment structure of the two possible host–endosymbiont pairs considered in our analyses. Each cell has a cytoplasm compartment [c] and can exchange compounds with its external environment (indicated by arrows). In endosymbioses, the extracellular compartment of the endosymbiont is the cytoplasm compartment of its host. (*B*) A bar graph with an inset of a violin plot shows the percentages of paired metabolisms that form viable endosymbioses for each of the three metabolic model collections. The percentages are the means of 100 samples of 1,000 pairs and are of similar magnitude, 56.2 to 66.2%; however, an ANOVA confirms the means do differ across collections (*P* < .001). (*C*) A bar graph shows the percentage of pairings in which neither, only one, or both configurations of endosymbioses are viable. There is at least one configuration viable for 84 to 92% of pairs in each collection and the most common scenario is where only one configuration is viable.

With our broad array of metabolic networks, we can assess the metabolic viability, of putative prokaryotic endosymbioses scenario (1) in Fig. 1. We considered 100,000 random pairs of networks sampled from each collection, which were split into 100 sets of 1,000 to estimate variation. For each pair of networks, we constructed two metabolic models of the endosymbiosis where networks swapped roles as host and endosymbiont (Fig. 2*A*). Importantly, every metabolic network in our dataset includes an environment—or equivalently a set of available extracellular compounds—that enable the organism (network) to grow. If we combine the environments from two metabolic networks, we obtain a joint environment in which both networks can grow independently. Thus, we can assess viability of an endosymbiosis by determining whether the host–endosymbiont system can grow in the joint environment (*Methods: Assessing Growth and Viability of Metabolisms*).

Fig. 2*B* shows that the average percentage of viable host–endosymbiont pairs varies between 56.2% and 66.2% across the three collections. An ANOVA confirms that the averages between collections are statistically different (*P* value < .001). Such differences may stem from many possible factors including model format, the set of reactions used, the particular compounds available in the environment, and/or the composition of species and their representative environments. We can observe other differences between collections if we evaluate factors that may predict whether an endosymbiosis is viable—such as similarity in reactions or biomass compounds between host and endosymbiont (*SI Appendix*, *Predicting Viability*). Yet, despite differences between collections, all 300 sets of samples have a viable percentage between 52 and 71%, indicating a similar scale for viability estimates. Moreover, if we consider both possible configurations of endosymbioses, we find that in each collection of metabolic models the majority of pairs, 84 to 92%, have at least one configuration that is viable (Fig. 2*C*), with most of those pairs (52 to 67%) having just one specific configuration that is viable.

**Fig. 3.**
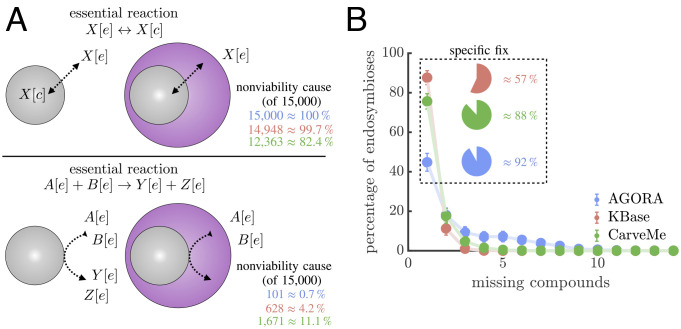
Paths to fixing nonviable endosymbioses. (*A*) A schematic shows the two causes of nonviability in endosymbioses using metabolic models: 1. (*Top*) missing transport of an extracellular compound into the host’s cytoplasm and 2. (*Bottom*) no access to compounds that remain in the extracellular compartment. For each, we show a typical form of the missing essential reaction and the percentage of 15,000 nonviable endosymbioses that can be made viable by providing the necessary type of reaction (colors indicate model collections, following the legend in *B*). Between the two causes, the first is a more frequent source of nonviability. Note that within a collection the percentages for the two causes do not sum to 100% because they are not mutually exclusive. (*B*) The graph shows the minimum number of compounds whose transport needs to be provided in order to fix nonviable endosymbioses. Each point is the mean of 100 samples of 100 fixable, nonviable endosymbioses, and the error bars are the standard deviations of those samples. For each collection, the most common case is that the endosymbiosis can be fixed by transporting a single compound. The pie chart inset shows how often that single compound is a specific compound, i.e., there is only one such compound whose transport makes the endosymbiosis viable.

Within the context of metabolic models, there are two nonexclusive reasons that host–endosymbiont pairs may be nonviable in an environment where each can survive separately (Fig. 3*A*). First, the host may lack a way of transporting a resource (or waste product) that the endosymbiont requires (or produces) in order to grow. Overcoming this cause of nonviability requires transport of a compound (or compounds) between the host’s cytoplasm and the extracellular environment. Such transport typically occurs in metabolic models via particular reactions, and so the host–endosymbiont pair could be made viable by gaining the appropriate transport reactions, which may occur in real populations via horizontal gene transfer. The second cause of nonviability stems from the endosymbiont needing to use a reaction whose compounds never enter the cell. Since the endosymbiont cannot directly access the external environment, it cannot perform the necessary reaction. This obstacle can only be overcome if the endosymbiont has direct contact between its membrane and the environment, which is infeasible based on the compartmental structure of an endosymbiosis. Thus, we focused on the first cause of nonviability and evaluated how easily host–endosymbiont pairs can be made viable.

We assessed how often nonviability stems from the host lacking a transport reaction by allowing hosts the ability to transport all compounds that exist in both cytoplasm and extracellular compartments. We attempted to repair 15,000 nonviable endosymbioses in each model collection and found that the majority can be made viable (100% for AGORA, 99.7% for KBase, and 82.4% for CarveMe). Although adding transport mechanisms for all compounds can fix the majority of nonviable endosymbioses, the likelihood that nonviability can be overcome depends on the number of transport mechanisms needed for viability. We estimated the minimum number of missing transport mechanisms by finding a set of compounds that fix viability only through their combined transport, i.e., failure to transport any one of them leads to nonviability. Using a series of linear programs (*Methods: Fixing Viability*) on 10,000 randomly sampled nonviable—yet fixable—endosymbioses from each model collection, we find that the majority of nonviable pairings can be fixed by the addition of a single transport mechanism (Fig. 3*B*). Yet, of those endosymbioses that can be fixed by a single transport mechanism, the majority (57 to 92%) require a specific compound without which they cannot be viable.

Even if a nascent endosymbiosis is viable, it still faces the challenge of competing against a background population composed of its direct ancestors. In principle, the competition can take many forms depending on the nature of selection. We first consider competition in terms of the ability of metabolisms to remain viable in the face of environmental degradation. Since host–endosymbiont pairs can draw on two sets of reactions, they may be better able to adjust to changing resource pools so as to have a survival advantage compared to their ancestors, increasing their probability of persistence (scenarios (2) and (3) in Fig. 1). We evaluated this possibility by randomly choosing 10,000 viable host–endosymbiont pairs and determining whether they remain viable when a single compound is removed from the environment. We investigated all possible environments where a single compound is missing, and in each modified environment we also assessed the viability of the ancestral host and endosymbiont metabolisms for comparison.

**Fig. 4.**
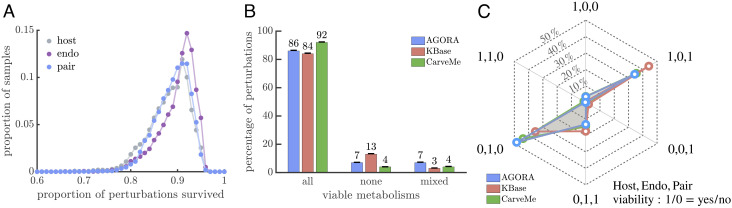
Survival competition between endosymbiosis and ancestral metabolisms in response to environmental degradation. (*A*) The distributions show the robustness of 10,000 endosymbiosis metabolisms (labeled “pair”) and their ancestral metabolisms (labeled “host” or “endo”) sampled from AGORA. Robustness is quantified as the proportion of environmental perturbations survived by a metabolism. The distributions have similar single peaked shapes with > 80% of the population lying between the range of 85 to 95%. *SI Appendix*, Fig. S3 shows a similar figure for the other collections. (*B*) The bar graph shows the percentage of environmental perturbations for which all three metabolisms are viable, nonviable, or mixed across the three model collections. In only 3 to 7% of perturbations is there a difference in the viability of the endosymbiosis metabolism compared to at least one of its ancestral metabolisms. (*C*) The star plot displays the relative frequency of the possible outcomes for the mixed cases from *B*. Of the 6 possible scenarios for mixed viability, the two most frequent feature different survival between the ancestral endosymbiont metabolism compared to the other two, though which is more robust depends on the model collection (*SI Appendix*, Fig. S4). The star plot also shows that endosymbioses survive more environmental perturbations than their ancestral host metabolisms in all collections.

We observe similar population-level patterns of survival in response to environmental degradation between the host–endosymbiont pair and its ancestral metabolisms (Fig. 4*A* and *SI Appendix*, Fig. S3). Furthermore, we find that the metabolisms act identically in 93 to 97% of perturbations, with the most frequent occurrence being that all survive (Fig. 4*B*). Of the 3 to 7% of environmental perturbations where we see differences in survival between metabolisms, three scenarios occur most frequently: 1) only the ancestral endosymbiont survives, 2) only the ancestral endosymbiont does not survive, and 3) only the ancestral host does not survive (Fig. 4*C*). As a consequence, the host–endosymbiont pair survives more environmental perturbations than the ancestral host metabolism in all model collections (*SI Appendix*, Fig. S4). If we instead compare the host–endosymbiont pair with the ancestral endosymbiont metabolism, the results depend on the collection such that host–endosymbiont pairs survive more often in KBase models while the reverse is true in AGORA and CarveMe (*SI Appendix*, Fig. S4).

**Fig. 5.**
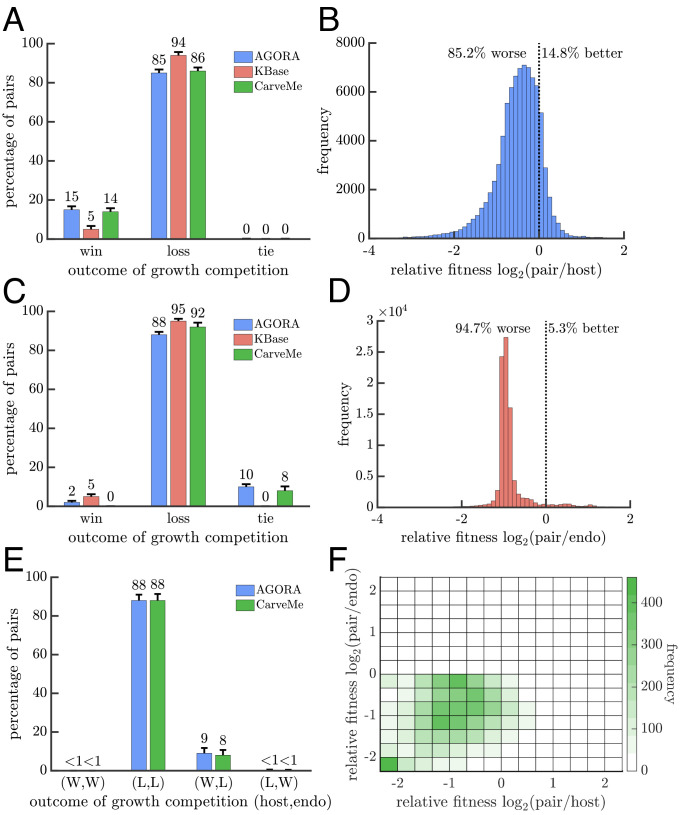
Growth-rate competition between endosymbioses and ancestral metabolisms. (*A*) The bar graph shows the result of a growth-rate competition between endosymbioses and their ancestral host metabolisms. The host grows faster for a majority of comparisons (85 to 94%). (*B*) The histogram shows the relative growth rates of endosymbioses versus their ancestral host metabolisms, sampled from AGORA (*SI Appendix*, Fig. S6 for KBase and CarveMe). When the endosymbiosis grows faster than its ancestral host, the fitness advantage is often smaller in magnitude compared to its fitness disadvantage when it grows more slowly. (*C*) The bar graph is similar to A but the comparison is between endosymbioses and their ancestral endosymbiont metabolisms. Again endosymbioses grow more slowly in the majority of comparisons (88 to 92%). (*D*) The histogram shows the relative growth rates of endosymbioses versus their ancestral endosymbiont metabolisms, sampled from KBase (*SI Appendix*, Fig. S7 for AGORA and CarveMe). As in *B*, the typical growth advantage is smaller in magnitude than the growth disadvantage for endosymbioses. (*E*) The bar graph shows the result of a growth-rate competition between the endosymbiosis and its ancestral metabolisms when all share the same environment. In both collections, the most likely scenario is that the endosymbiosis grows more slowly than both ancestral metabolisms (88%). (*F*) The relative fitness of the endosymbiosis versus its ancestral metabolisms from *E* is plotted using CarveMe models (*SI Appendix*, Fig. S8 for AGORA).

In addition to survival selection, genome-scale metabolic models enable us to consider competition in terms of population growth rates. Instead of simply evaluating a binary outcome of viable versus nonviable, we can use a metabolism’s computed maximum rate of biomass production as a proxy for its population-level growth rate. Actual growth rates may depend on many additional factors related to the physiological state of cells, e.g., gene regulation, or the state of the environment, e.g., temperature. Properly accounting for such factors requires adding extra constraints or structure to the metabolic models, often informed by data from supplemental experiments. Since we lack the necessary information for the vast majority of metabolic models and imposing additional constraints or structure may introduce new sources of inaccuracies, we used the default versions of the metabolic models. However, to limit the influence of issues concerning the absolute accuracy of growth rate predictions, we consider only their relative values by comparing the endosymbiosis to its constituent metabolisms. With this approach, we compute the fitness of viable host–endosymbiont pairs and find that they grow slower than both their ancestral host and endosymbiont metabolisms in over 85% of samples from each model collection (Fig. 5*A*–*D*). In the rare cases where the host–endosymbiont pair grows faster than its ancestors, its average growth-rate advantage is smaller in magnitude than its average disadvantage when the ancestral metabolisms grow faster. For example, in AGORA models when the host–endosymbiont pair grows faster than the ancestral host metabolism, its mean advantage is 18.9% relative to the host, but when the ancestral host metabolism grows faster, its mean advantage is 66.9% relative to the host–endosymbiont pair.

Since an endosymbiosis has to sustain two metabolisms, it could be that comparing its growth rate to those of an isolated ancestral host or endosymbiont metabolism is not a fair comparison. We address this possible issue by computing the growth rates of all three metabolisms when grown together in the same environment (*Methods: Growth Rate Calculation of Communities*). In short, we maximize each metabolism’s growth rate assuming that the total flux of metabolites through the system is maximal. We restrict our analyses to AGORA and CarveMe models because KBase models often yield multiple possible growth rates. Fig. 5*E* shows that in the majority of cases (88% of samples) the host–endosymbiont pair grows more slowly than both ancestral metabolisms. It is rare (< 1%) that the host–endosymbiont pair can grow faster than the ancestral endosymbiont metabolism. In the 8 to 9% of samples where the host–endosymbiont pair grows faster than its ancestral host metabolism, scenarios (2) and (3) in Fig. 1, the growth rate advantage is smaller in magnitude than its concomitant disadvantage compared to the ancestral endosymbiont metabolism (Fig. 5*F*).

Although endosymbioses grow more slowly than their ancestors, they may be competitive if they are more evolvable. We evaluate this possibility by determining the effects of mutations on metabolisms when they are independent or in an endosymbiosis (*Methods: Evolvability Assessment*). We focus on mutations that increase the bounds on reaction fluxes, which will either have no effect on a metabolism’s growth rate (neutral mutations) or increase it (beneficial mutations). We randomly sample 1,000 endosymbioses that grow more slowly than their ancestors and compute the effects of all possible single mutations in host and endosymbiont reactions. We identified beneficial mutations in AGORA and CarveMe models but did not find any in KBase models because reaction bounds are not the dominant constraint on growth rates in KBase. The evolvability analyses will thus rely only on models from the AGORA and CarveMe collections, though we did find similar results in KBase after imposing reaction bounds that do limit growth (*Methods: Evolvability Assessment*).

**Fig. 6.**
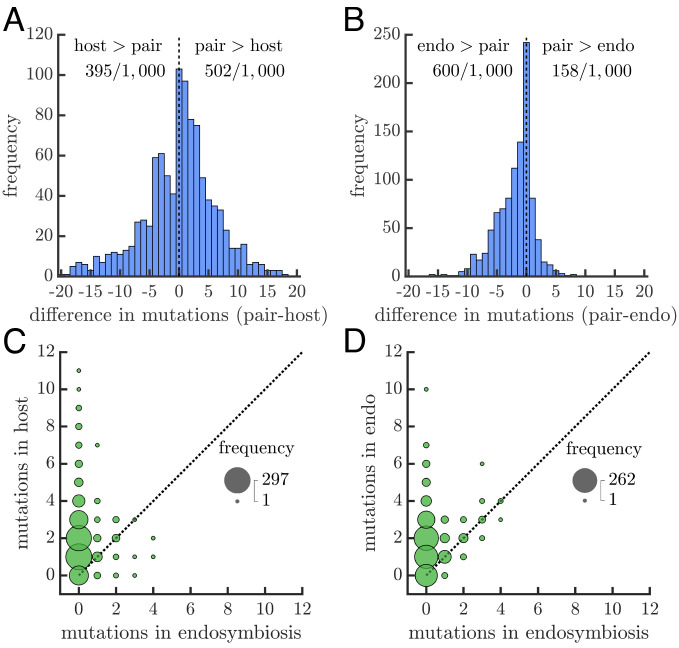
Evolvability of endosymbioses versus ancestral metabolisms. (*A*) The histogram shows the difference in the number of beneficial mutations between the endosymbiosis and its ancestral host metabolism (data for A & B from AGORA, *SI Appendix*, Figs. S9 and S10 for CarveMe). All mutations occur in host metabolism reactions and are deemed beneficial if they increase the growth rate. A sign-rank test supports the null hypothesis that the endosymbiosis and its ancestral host metabolism do not differ in the number of beneficial mutations. (*B*) The histogram is similar to A but compares the endosymbiosis to its ancestral endosymbiont metabolism for mutations in their shared reactions. Here, a sign-rank test rejects the null hypothesis, such that the ancestral endosymbiont metabolism has more beneficial mutations (*P* < .01). (*C*) A bubble chart shows the frequency of mutations, in endosymbioses vs. their ancestral host metabolisms, that increase the growth rate above that of the ancestral host metabolism (data for *C* & *D* from CarveMe, *SI Appendix*, Figs. S11 and S12 for AGORA). There are more such mutations in ancestral host metabolisms than in endosymbioses. (*D*) The plot is similar to *C* except it is in relation to the ancestral endosymbiont metabolism. Again, there are more such mutations in ancestral endosymbiont metabolisms than in endosymbioses.

If we consider only mutations in host reactions, we find that there is no statistical difference in the number of beneficial mutations in an endosymbiosis compared with its ancestral host metabolism (Wilcoxon signed rank test, *P* ≈.07 in CarveMe and *P* ≈.27 in AGORA, Fig. 6*A*). In contrast, mutations in endosymbiont reactions are less often beneficial in an endosymbiosis than in its ancestral endosymbiont metabolism (Wilcoxon signed rank test, *P* < 10^−6^ in both CarveMe and AGORA, Fig. 6*B*). Of the mutations that are beneficial in an endosymbiosis, few increase the growth rate beyond those of the ancestral metabolisms—only 5.0 to 5.6% of beneficial mutations in host reactions and 17.0 to 18.1% in endosymbiont reactions. Moreover, the majority of endosymbioses do not have any mutations that increase their growth rate above their ancestral metabolisms (Fig. 6 *C* and *D*). It could be possible, though rare, that an endosymbiosis may have access to mutations that offer it greater growth benefits than its ancestral metabolisms. We evaluated this possibility by comparing the maximal growth rate reached by mutations in the endosymbiosis versus its ancestral metabolisms and found no such example where the endosymbiosis obtains the highest fitness.

## Discussion

An endosymbiosis between two prokaryotes gave rise to eukaryotes and likely facilitated the evolution of large complex organisms, but this is one of only a few examples of prokaryotic endosymbioses. While studies have identified possible obstacles to the formation of prokaryotic endosymbioses, there is a lack of quantitative frameworks that allow us to estimate their influence. Here, we introduce a quantitative framework using genome-scale metabolic networks that evaluates the role of metabolic compatibility in the different stages of prokaryotic endosymbiosis evolution: viability, persistence, and evolvability. We find that over half of random pairs of metabolisms can form viable endosymbioses; however, the resulting endosymbioses often face fitness costs in terms of growth that they are unlikely to overcome through mutations.

A major result of our work here is that in the first stage toward a successful endosymbiosis—initial viability—metabolic compatibility produces only a very limited barrier, with over half of endosymbiotic configurations being viable (avoiding scenario (1) in Fig. 1). On the one hand, this is surprising given the substantial biochemical and metabolic diversity of prokaryotes. On the other hand, all cells share a metabolic core (e.g., the TCA cycle), which could facilitate the compatibility of metabolic networks (35–37). Indeed, we found that when endosymbionts and hosts share a greater fraction of metabolic reactions, they are more likely to produce viable endosymbioses (*SI Appendix*, Fig. S2). Certain ecoevolutionary processes may act to increase the frequency of successful metabolic pairings, such as the frequent exchange of entire metabolic pathways via horizontal gene transfer (38–40) or the transmission of plasmids in bacterial communities (41). Even in the absence of any history of gene sharing, syntrophy often emerges spontaneously when metabolic networks are coupled (42). Since over 84% of the species pairs we considered had at least one viable configuration, the small barrier to metabolic viability seems to reflect more the spatial structure of paired metabolic networks than the composition of the networks per se.

Our analysis of metabolic viability was averaged across hundreds of species in different model collections so there may be important factors that influence viability that were masked. For example, many prokaryotes have remarkably sophisticated ecto symbioses, such as aggregates of archaea and bacteria that perform anaerobic methane oxidation by passing electrons extracellularly (43, 44), the many close association symbioses in biofilms and microbial mats (45, 46), and the ecosystems defined by effective supermetabolisms (47). We may expect that the particular ecological interaction between a pair of prokaryotes, or their taxonomic classification, may affect the probability they can form a viable endosymbiosis (48). In terms of eukaryogenesis, a prevalent theory is that the host was an archaean and the endosymbiont was a bacterium, and the two species may have had an established syntrophic interaction prior to forming the endosymbiosis (49). The collections we used for our analyses do not contain enough data to allow us to adequately assess the relative likelihood of such an event. However, the limited data we do have indicates that there may be complex interactions between such factors (*SI Appendix*, sections “*Archaea-bacteria pairings*” and “*Ecological interactions and viability*”).

While metabolic compatibility did not provide a significant challenge to viability, it did emerge as a substantial filter during the persistence and evolvability stages, when endosymbioses compete with their ancestral metabolisms. Whether metabolisms competed to grow fastest or survive environmental perturbations, scenarios (4) and (5) in Fig. 1, an endosymbiosis rarely held a competitive advantage over both of its ancestors (< 1% of cases). In addition, mutations in endosymbioses rarely produced a growth advantage over ancestral endosymbionts (17 to 18.1%, Fig. 6*D*) or ancestral hosts (5 to 5.6%, Fig. 6*C*). These outcomes may be surprising given that all-else-being-equal increased network size should confer increased robustness to environmental perturbations, increased growth rates, and increased capacity to adapt (5, 6, 50). However, unlike their ancestral metabolisms, endosymbioses must satisfy the biomass requirements of two metabolisms. Within this context, it is then somewhat expected that an early endosymbiosis may experience a fitness disadvantage compared to its ancestors, especially in the absence of any mechanism to coordinate or divide labor. Furthermore, the need to satisfy two sets of biomass requirements can limit or mask the effects of beneficial mutations, making it difficult to overcome initial fitness disadvantages.

Examining the rare cases when an endosymbiosis has higher fitness than its ancestors can shed light on the conditions in which it can persist. When subjected to environmental perturbations, the endosymbiosis has a competitive advantage against its ancestral host species. Similarly, when a premium is put on fast growth, the endosymbiosis is more likely to outcompete its ancestral host species than the ancestral endosymbiont species. Taken together, these results suggest that if an endosymbiosis were able to persist, it would likely replace its ancestral host. This result is supported by the current ecological distributions of microbial eukaryotes and prokaryotes. Microbial eukaryotes coexist in abundance with the ancestral lineage of their mitochondrial endosymbiont, the alpha-proteobacteria (51, 52), but exhibit much more limited coexistence with the ancestral lineage of the host, presumably of archaeal origin—likely a Lokiarchaeota (53–56).

Our competition analyses also suggest that while an endosymbiosis almost never grows faster than both of its ancestors, an appropriate combination of conditions that favor *K*-strategists may allow a nascent endosymbiosis to survive when both its ancestors do not. These results are consistent with the argument that compared to bacteria, eukaryotes may have been *K*-selected with slower rates of reproduction and stable resource supplies (57). The results are also corroborated by the observation that the few other documented prokaryotic endosymbioses are found in nutrient-stable environments (15, 16, 58), e.g., the bristles of a scale worm near a deep hydrothermal vent (58) or in a specialized organ of a sap-feeding mealybug (16). Regarding the origins of eukaryotes, it is difficult to resolve the relevant environmental drivers, but recent work indicates that the Great Oxidation Event may have played a role (59). During this period when sulfate was suddenly more available, an endosymbiosis, in the form of a hydrogenosome, may have granted methanogens an endogenous supply of hydrogen in the face of sulfate reducers that soak up environmental sources (60). Although these arguments are about a growth advantage for early eukaryotes, rather than a K-selection advantage (57), they may explain the sudden radiation of endosymbioses to high abundance. Together, these various arguments and observations suggest that particular environmental contexts or shifts may have played an important role in the origin and establishment of the eukaryotic endosymbiosis—though explaining why only extremely rare environments or periods lead to prokaryotic endosymbioses requires further study.

Throughout this paper, we have made general predictions about the role of metabolic compatibility in shaping nascent prokaryotic endosymbioses, drawing conclusions from three different metabolic network collections. Although the results from each collection broadly agree about many aspects of metabolic compatibility in endosymbioses, there are some key differences. For example, the outcome of survival competition between a host–endosymbiont pair and its ancestral endosymbiont metabolism depended on the collection (*SI Appendix*, Fig. S4). Such differences could stem from many factors including the organisms, environments, or procedures used to construct the networks themselves. Furthermore, the completeness of metabolic models is also an issue as it is likely that many metabolic models are missing reactions and processes. Research into improving metabolic network construction will likely lead to much future development. While we expect such development to produce refinements in our quantitative predictions, it is unlikely to change the broader conclusions that were common across the diverse collections considered here. For instance, our results show that compatibility between metabolisms is common, so if future development in metabolic models adds more reactions, it will likely only lead to increased compatibility, i.e., our results represent a lower bound estimate for metabolic compatibility. Regardless of our expectations, our metabolic endosymbiosis framework could readily accommodate future advances in the field of metabolic networks; it simply requires rerunning the computations with updated networks or different environmental conditions. Thus, our quantitative framework is adaptable and should continue to make useful predictions concerning the role of metabolic compatibility in the evolution and ecology of prokaryotic endosymbioses.

An overarching goal of our work has been to assess factors responsible for the relative rarity of prokaryotic endosymbioses. We have focused on metabolic compatibility because it was amenable to existing methodology and plays a role in the initiation, persistence, and evolvability of endosymbioses. Though our results reveal significant limitations imposed by metabolic compatibility, it is unlikely that metabolic compatibility alone accounts for the relative rarity of prokaryotic endosymbioses. If it were the dominant barrier, then the relative abundance of eukaryotic endosymbioses would imply that metabolic compatibility is far less constraining when the host is a eukaryote. We can gain some insight into this possibility by considering the abundant examples of endosymbioses with eukaryotic host cells (61, 62). We expect that multicellular eukaryote hosts can more easily accommodate intercellular endosymbionts because they can manipulate the space between their cells to create specialized metabolic environments (63), such as light organs in bobtail squid (64, 65) or hindguts in termites (66). If, instead, we consider only intracellular endosymbionts which can be found in both multicellular and unicellular eukaryotes (12, 61, 62, 67–69), then many of these also involve specialized spaces, e.g., bacteriocytes (70) or vacuoles (11). The fact that so many eukaryotic endosymbioses involve specialized compartments suggests some adaptive benefit to controlling the metabolic exchanges between species, e.g., they may not be innately synergistic, though it is unclear whether these spaces evolved prior to or in conjunction with the endosymbiosis.

The frequent presence of structured compartments for endosymbionts may indicate a major barrier to establishing a persistent, evolvable prokaryotic endosymbiosis. In the absence of specialized compartments, our analyses show that problems with metabolic transport are the primary causes of nonviability. Introducing additional structures that separate organisms without appropriate modes of transport would likely increase the risk of a nonviable endosymbiosis. If these structures should capture some of the spatial complexity of metabolism in prokaryotic communities featuring ectosymbioses (43, 44, 71–76), then they would require complex physiological and morphological remodeling of the host cell. Here, eukaryotes may have an advantage compared to prokaryotes as they share a common set of complex basal traits including energy-producing mitochondria, dynamic cytoskeletons, endomembrane systems, a nuclear structure, and meiotic sex (1, 8, 77). Together, these traits could better equip eukaryotes to evolve novel cellular environments and structures and thus explain why subsequent endosymbioses are relatively common in eukaryotes—i.e. because they have already solved the key issues. In the context of internal structures and surfaces, it should be noted that while our modeling approach explicitly deals with a generalized geometric nesting of reactions (Fig. 2), there is much to be done in the future to add the explicit spatial physics of electron transfers and spatial metabolic fluxes within the cell.

Along with the barriers associated with dramatic internal physiological remodeling, it is also important to consider how two prokaryotes actually form an endosymbiosis. Here, an often invoked barrier to prokaryotic endosymbioses is the difficulty of internalizing a cell within another in the absence of phagocytosis, or a similar mechanism of engulfment (17). Indeed, the ubiquity of phagocytosis in eukaryotes creates significantly more opportunities to initiate an endosymbiosis, e.g., amoebas frequently engulf different bacteria that can resist digestion (78). Yet, phagocytosis may not be the only physiological mechanism for a cell to internalize another cell (e.g., refs. 17, 79 and 34). Many other studies have demonstrated the plasticity of prokaryote membranes and cytoskeletal architecture (53, 80–82), suggesting the relative absence of phagocytosis may not be as dominant a barrier as once thought. And the continuing discovery of new functions, traits, and morphologies in prokaryotes also emphasizes the importance of evaluating multiple barriers, not just phagocytosis. Indeed, remarkably, phylogenetic analysis of a prokaryote endosymbiosis within mealybug cells suggests that the endobacterial symbiosis may have originated multiple times during mealy bug diversification (16), despite the prokaryote host lacking an obvious capacity for endocytosis. It thus seems highly probable that many more similar examples of such endobacteria symbionts remain to be discovered, especially given that this prokaryotic endosymbiosis was previously overlooked (83) and the microbiomes of a million or more invertebrate species (84) have not been investigated. Moreover, a low barrier to initiation and viability of prokaryotic endosymbioses suggests that the observed acquisition by all major archaea of numerous bacterial genes (85) could reflect, among other things, numerous incipient endosymbioses that facilitated interdomain gene transfer but ultimately went extinct due to low persistence and evolvability.

Ultimately, given the functional diversity and abundance of microbes on Earth, the expanding list of discoveries that soften phagocytosis as a barrier, and the metabolic viability of many potential endosymbiosis, a reasonable interpretation of our results is that hundreds to thousands or more prokaryote endosymbioses remain to be discovered. However, the reduced metabolic robustness, growth rates, and evolvability that these endosymbioses likely face severely limits their persistence and potential to radiate spectacularly. The high rates of extinction and low rates of speciation resulting from these metabolic considerations would sustain only a relatively low diversity of extant prokaryotic endosymbioses confined to environments in which they are less likely to be outcompeted by ancestral host or endosymbiont lineages, such as nutrient-poor environments.

## Materials and Methods

### Genome-Scale Metabolic Model Curation.

We obtained metabolic models of diverse prokaryotes in .xml format made available in ref. 86 for AGORA (87), KBase (88), and CarveMe (89) collections (*SI Appendix*, Table 1 for details). We used the Constraint-Based Reconstruction and Analysis Toolbox (COBRA) (90) to create .mat files for further analysis using MATLAB (91). The .mat format of a metabolic model includes a stoichiometric matrix of compounds and reactions, a list of compound names, lower and upper bounds for reaction fluxes, an objective function that identifies the biomass reaction, a right-hand-side vector that indicates how each compound is balanced, and other identifying information. Metabolic models are partitioned across two compartments (cytoplasm [c] and extracellular [e]) except those in CarveMe which include an additional periplasm [p] compartment. All models come with an implicit environment—determined by the bounds on reaction fluxes and right-hand-side vectors—that make a set of extracellular compounds accessible so the metabolism is viable, i.e., it can synthesize all of its biomass compounds. Determining whether a metabolism is viable requires performing flux balance analysis and solving the associated linear program. We used the Gurobi optimization software (92) to solve all linear programs and confirmed that all metabolic models are initially viable, where viability is defined as having an objective function value (growth rate) above a tolerance of 0.001.

For each model collection, we restructured the metabolic models to facilitate analyses of possible endosymbioses. First, we identified all compounds and reactions used within a metabolic model collection and reformatted the stoichiometric matrices (*S*) so that the same row corresponds to the same compound across models. Second, we changed how environmental compounds are made available to metabolic models. Initially, metabolic models access environmental compounds through source/sink reactions whose upper and lower bounds on flux determine how much of the compound is available. We replaced these reactions with mathematically equivalent constraints on the amount of compound available. So, a source/sink reaction flux *r*_*i*_ for compound *c*_*j*_ with lower and upper bounds *l*_*i*_ and *u*_*i*_, respectively, would be replaced with the equivalent constraint on the derivative of compound concentration, $l_{i}\leq c_{j}\prime \leq u_{i}$, note $c_{j}\prime =\sum_{k}S_{j,k}r_{k}$. This modification makes it easier to create joint environments using different metabolic models. Finally, for each metabolic model, we created different stoichiometric matrices depending on its role as either host or endosymbiont. When the metabolism is a host, its stoichiometric matrix (*S*_*H*_) is the same as if it were growing in isolation. When the metabolism is an endosymbiont, its extracellular compartment is identical to the cellular compartment of the host. Thus, we partitioned the stoichiometric matrix depending on whether the compounds are strictly within the endosymbiont (*S*_*E*_) or within the host (*S*_*E* → *H*_).

### Assessing Growth and Viability of Metabolisms.

We compute the growth rate of metabolisms, either in isolation or in endosymbioses, by performing flux balance analysis and solving the associated linear program. If the index for the biomass reaction is *λ* then the linear program for its growth in isolation is:

$$\begin{array}{c} \begin{array}{cc} \;\text{maximize} & x_{\lambda }\\ \;\text{subject to} & \bar{a}\leq S_{H}\bar{x}\leq \bar{b}\\ \;\;\; & \bar{l}\leq \bar{x}\leq \bar{u}, \end{array} \end{array}$$

where $\overset{¯}{x}$ is a vector of reaction fluxes, $\overset{¯}{l}$ is a vector of lower bounds for fluxes, $\overset{¯}{u}$ is a vector of upper bounds for fluxes, *S*_*H*_ is the stoichiometric matrix, $\overset{¯}{a}$ is a vector of lower bounds for derivatives of compound concentrations, and $\overset{¯}{b}$ is a vector of upper bounds for derivatives of compound concentrations.

For an endosymbiosis, we denote the fluxes of the host as $\overset{¯}{x}$ and those of the endosymbiont as $\overset{¯}{y}$. We use the *H* and *E* indices to indicate host or endosymbiont, respectively. The linear program for two metabolisms in an endosymbiosis is then

$$\begin{array}{ll} \text{maximize} & x_{\lambda_{H}}\\ \text{subject to} & \bar{a_{H}}+\bar{a_{E}}\leq S_{H}\bar{x}+S_{E\to H}\bar{y}\leq \bar{b_{H}}+\bar{b_{E}}\\ & S_{E}\bar{y}=0\\ & \bar{l_{H}}\leq \bar{x}\leq \bar{u_{H}}\\ & \bar{l_{E}}\leq \bar{y}\leq \bar{u_{E}}\\ & x_{\lambda_{H}}=y_{\lambda_{E}}. \end{array}$$

The $S_{E}\overset{¯}{y}=0$ condition stems from the fact that compounds in the cytoplasm and periplasm compartments are balanced in the endosymbiont. The last condition *x*_*λ*_*H*__ = *y*_*λ*_*E*__ requires that the host and endosymbiont grow at the same rate. We note that this linear program corresponds to an early, primitive endosymbiosis prior to any adaptations; thus, it lacks much of the additional structure found in metabolic models of modern eukaryotes with established endosymbioses, e.g., ref. 93.

### Fixing Viability.

We attempted to fix nonviable endosymbioses through a multistep process. First, we determined whether the endosymbiosis could be viable if all transport between the cytoplasm and extracellular compartments are available. We identified all environmental compounds that 1) are available in the environment with nonzero amounts, 2) exist in both environmental and cellular compartments, and 3) are usable by the endosymbiont such that at least one reaction has it. We then added extra reactions in the host–endosymbiont joint stoichiometric matrix that enabled those compounds to be transported between the host’s [e] and [c] compartments. We performed flux balance analysis to determine the growth rate. If the growth rate was below the tolerance 0.001, it meant that the lack of viability was also due to the endosymbiont not having access to the external environment. We then confirmed that viability could be restored by providing the endosymbiont access to the external environment through creation of source/sink reactions in *S*_*E*_ for compounds present in the environment at nonzero amounts. All nonviable endosymbioses could be fixed by a combination of restoring transport and allowing the endosymbiont to access extracellular compounds. In cases where viability could be restored by providing transport reactions alone, we estimated a potential minimal set of compounds following the algorithm outlined in *SI Appendix*, Fig. S5 which is similar to the algorithm in (94).

### Growth Rate Calculation of Communities.

We compute the growth rates of the host–endosymbiont pair together with its ancestral metabolisms in the same environment through a two-step process. First, we compute the maximal flux through the entire community by maximizing the sum of fluxes through the three biomass reactions: ancestral host *x*_*λ*_*H*__, ancestral endosymbiont *x*_*λ*_*E*__, and host–endosymbiont pair *x*_*λ*_*P*__. Second, we maximize the flux through each of the three biomass reactions one at a time while requiring the community flux be held constant at its maximal value (call it *z*), i.e., we add the constraint *x*_*λ*_*H*__ + *x*_*λ*_*E*__ + *x*_*λ*_*P*__ = *z*. We perform the second step because the solution to a linear program may not be unique, so there may be multiple partitions of growth within a community that give rise to the same total community flux. For our analyses, we considered only those cases in which the growth rates of the host–endosymbiont pair and its ancestral metabolisms are unique when the total community flux is maximal. We determined uniqueness by requiring that the computed growth rates for metabolisms did not vary above 10% between its highest and lowest values. We found that the AGORA and CarveMe collections frequently gave unique values, 99.7% and 98.2% of computations, respectively; however, KBase only met this criterion for uniqueness in 0.02% of computations. Despite the lack of uniqueness in KBase-computed growth rates, the maximal host–endosymbiont pair growth rate rarely exceeded the growth rates of both its ancestral metabolisms, 3 out of 10,000 computations.

### Evolvability Assessment.

We evaluate the adaptive potential of metabolisms by determining how the computed growth rate is affected by mutations that increase the bounds of reactions. Since increasing the bounds of reactions can never decrease growth rates, the mutations are either neutral or beneficial. For ancestral metabolisms, we systematically modify each reaction bounds one at a time by a factor of 1000, effectively removing that reaction as a limiting constraint. Thus, if a reaction flux *x*_*i*_ has a lower bounds *l*_*i*_ (where *l*_*i*_ ≤ 0) and an upper bound *u*_*i*_, then we transform its bounds to −1000|*l*_*i*_|≤*x*_*i*_ ≤ 1000*u*_*i*_. We compute the growth rate following this mutation and then reset the bounds to evaluate another mutation. For host–endosymbiont pairs, we evaluate both mutations in endosymbiont reactions and host reactions separately.

While our methodology to assess evolvability was able to identify mutations that affected the growth rate in AGORA and CarveMe collections, it did not find any in KBase. The lack of any beneficial mutations in KBase suggests that the reaction bounds do not constrain the growth rate. We tested this hypothesis by decreasing the bounds of all reaction rates by a factor of 100 and recomputing growth rates. We found that the growth rates did decrease and we could identify beneficial mutations in the metabolisms of the host–endosymbiont pair as well as its ancestors. The results are similar to the analyses with AGORA and CarveMe described in the main text: Mutations were more often beneficial in ancestral metabolisms than host–endosymbiont pairs (87.7% for host reactions and 78.0% for endosymbiont reactions).

## Supplementary Material

Appendix 01 (PDF)

## Data Availability

Matlab files data have been deposited in github (95).
