# Supplementary material for: Metabolic compatibility and the rarity of prokaryote endosymbioses
Source: Proc Natl Acad Sci U S A. 2023 Apr 18;120(17):e2206527120. doi: 10.1073/pnas.2206527120 (PMC10151612; doi:10.1073/pnas.2206527120)
Supplement: Supplementary file 1 — Appendix 01 (PDF) [file pnas.2206527120.sapp.pdf]

Supplementary Material

Metabolic model collection information

Collection	Number of metabolisms	Number of compounds	Number of reactions
AGORA	818	2072	3815
KBase	1637	1675	3245
CarveMe	5587	2340	4245

Table S1: Summary data for metabolic model collections. AGORA contains metabolic reconstructions of human gut bacteria comprising over two hundred genera [1]. KBase is a platform created by The United States Department of Energy for sharing, integrating, and analyzing data of communities of plants and microbes [2]. CarveMe is a tool to automate the construction of metabolic models and circumvent issues with manual curation [3]. Together these three collections represent a large and varied set of metabolic models.

Predicting viability

In this section, we assess whether simple heuristics concerning the similarity of host and endosymbiont metabolic networks may predict viability. We first turn to the biomass reactions which effectively list all of the compounds required by the host and endosymbiont in order to grow. If the biomass reactions contain completely different compounds then we might expect a potential endosymbiosis to be nonviable because the host and endosymbiont have different metabolic needs. For example, if the endosymbiont requires a compound that the host does not need then the host may lack the ability to transport it from the external environment into its cytoplasm where the endosymbiont can access it. In contrast, if the biomass reactions contain the same compounds then both metabolisms need to make the same products, which would increase the likelihood that the host transports compounds needed by the endosymbiont. We predict then that the number of compounds from the endosymbiont's biomass reaction that are missing in the host, may increase the likelihood of nonviability. Figure S1 shows that models from AGORA match this expectation to some extent—though the relationship is not strictly monotonic. In KBase models the probability of viability is actually higher if one compound is missing rather than none. In CarveMe models, all biomass reactions contain the same compounds so the metric of missing compounds is not useful in predicting nonviability.

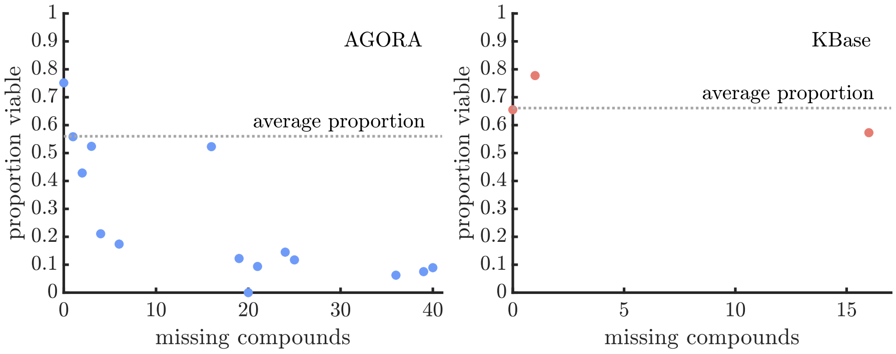

Figure S1: **Proportion of missing biomass compounds as an indicator of nonviability**. Each plot shows the proportion of endosymbioses that are viable as a function of the number of compounds in an endosymbiont's biomass reaction that are missing from the host's. The dashed line in each plot shows the average proportion of viable endosymbioses across all samples within a collection of metabolic models. The AGORA plot suggests a relationship between the number of missing biomass compounds and nonviability. KBase biomass reactions belong to a few distinct classes which makes it difficult to discern any relationship. Indeed the pairs of metabolisms with one missing biomass compound are more viable than those with none. All CarveMe biomass reactions contain the same compounds so there are no missing compounds.

Next we consider whether the proportion of reactions shared between the host and endosymbiont is a predictor of viability. If the host and endosymbiont share many reactions then they may also share metabolic pathways and higher order metabolic structures, making them more likely to be metabolically compatible. Figure S2 shows the results of this analysis. The relationships vary across model collections but once a host metabolism has $\geq 75\%$ of an endosymbiont's reactions there is an above average probability that they can form a viable endosymbiosis—although in KBase and CarveMe this relationship is not monontonic and even shows a dip when the host's metabolism contains all of the endosymbiont's reactions.

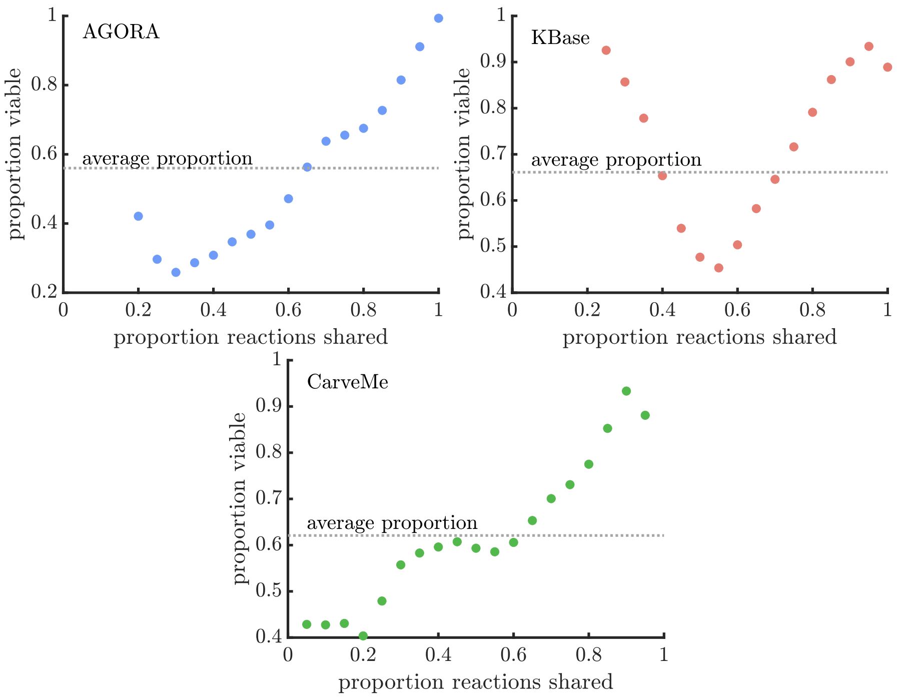

Figure S2: **Proportion of shared reactions as an indicator of viability**. Each plot shows the proportion of endosymbioses that are viable as a function of the proportion of an endosymbiont's reactions that are shared with the host. The dashed line shows the average proportion of viable endosymbioses across all samples within a collection. The plots show that the relationship between shared reactions and viability is not consistent across collections, e.g. KBase has a U-shaped relationship while CarveMe has a mostly monotonic relationship. One common feature is that if a host metabolism has $\geq 75\%$ of the endosymbiont's reactions then there is a higher than average probability that the resulting endosymbiosis is viable.

Finally, we note that if we use the same metabolic network for both host and

endosymbiosis we still find nonviable pairings: 7/818 (or 0.86%) in AGORA, 211/1637 (or 12.89%) in KBase,and 459/5587 (or 8.22%) in CarveMe.

Viability selection

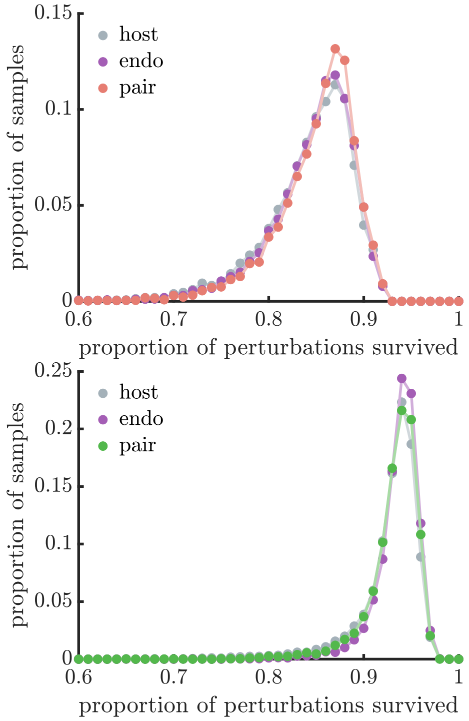

Figure S3: **Companion to Figure 4A.** The distributions show the robustness of 10,000 endosymbiosis metabolisms and their ancestral metabolisms sampled from KBase (top) and CarveMe (bottom). As in Figure 4A, robustness is quantified as the proportion of environmental perturbations survived by a metabolism. The distributions are similar in shape within metabolic model collections.

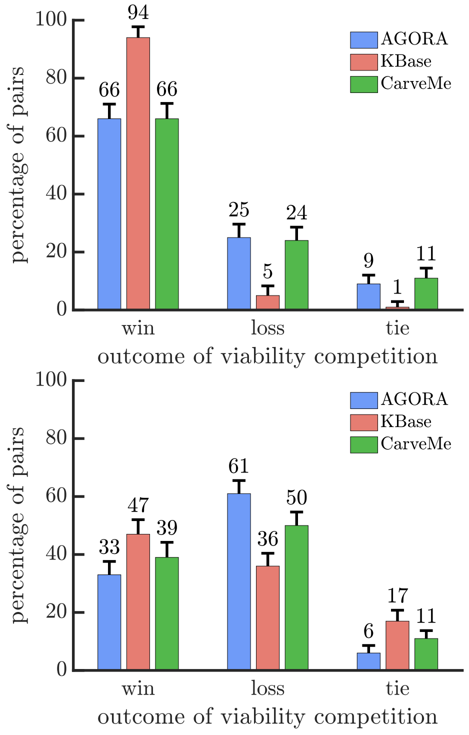

Figure S4: **Companion to Figure 4C.** (top) The bar graph shows the results of comparing the endosymbiosis with the ancestral host metabolism in terms of surviving environmental degradation. Across collections the endosymbiosis is more robust to environmental degradation than the ancestral host metabolism. (bottom) Similar to the top plot except it compares the endosymbiosis to the ancestral endosymbiont metabolism. The endosymbiosis is more robust than the ancestral endosymbiont metabolism in KBase models and less robust in AGORA and CarveMe models.

Flow chart

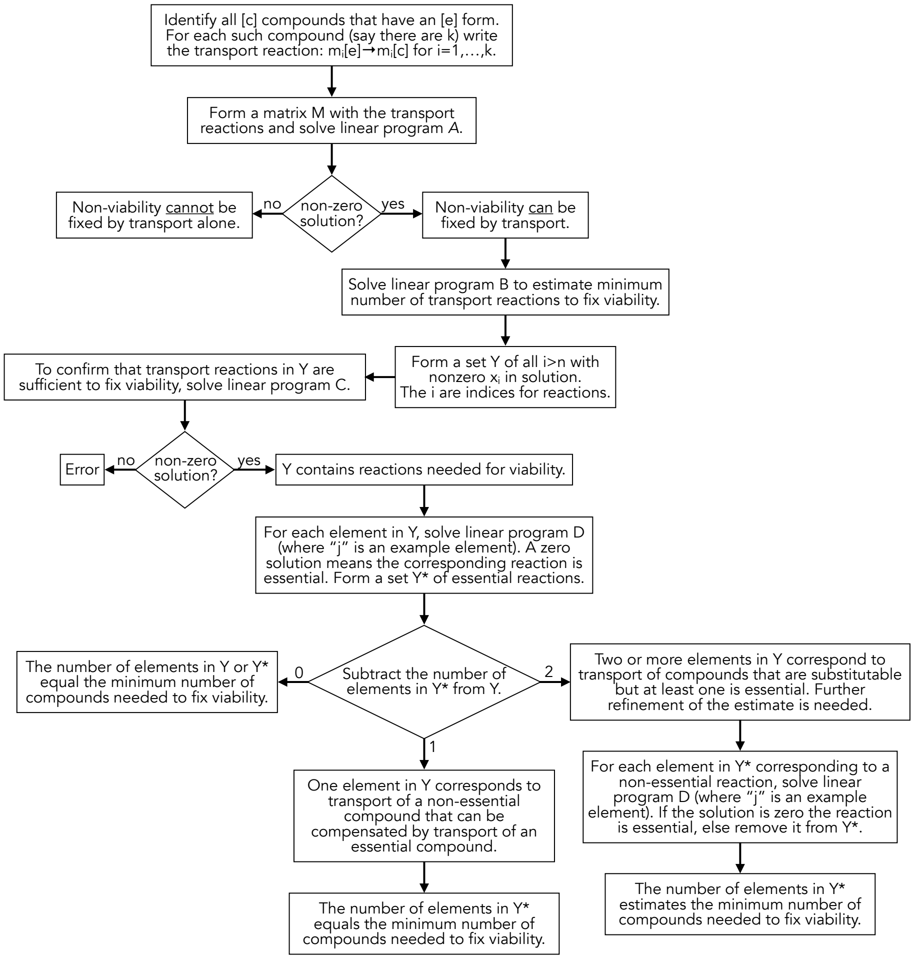

Linear programs

Original.

$$\text{maximize}: x_b$$
$$\text{subject to}:$$
$$\bar{a} \le S \le \bar{b}$$
$$l_i \le x_i \le u_i, \text{ for } i = 1, \ldots, n$$

A.

$$\text{maximize}: x_b$$
$$\text{subject to}:$$
$$\bar{a} \le S^* \le \bar{b}$$
$$l_i \le x_i \le u_i, \text{ for } i = 1, \ldots, n$$
$$0 \le x_i \le 10^3, \text{ for } i = n+1, \ldots, n+2k$$
$$\text{where}:$$
$$S^* = [S\ M\ (-M)]$$

B.

$$\text{minimize}: \sum_{i=n+1}^{n+2k} x_i$$
$$\text{subject to}:$$
$$\bar{a} \le S^* \le \bar{b}$$
$$l_i \le x_i \le u_i, \text{ for } i = 1, \ldots, n$$
$$0 \le x_i \le 10^3, \text{ for } i = n+1, \ldots, n+2k$$
$$x_b \ge 10^{-3}$$
$$\text{where}:$$
$$S^* = [S\ M\ (-M)]$$

C.

$$\text{maximize}: x_b$$
$$\text{subject to}:$$
$$\bar{a} \le S^* \le \bar{b}$$
$$l_i \le x_i \le u_i, \text{ for } i = 1, \ldots, n$$
$$x_i = 0, \text{ for } \{i > n | i \notin Y\}$$
$$0 \le x_i \le 10^3, \text{ for } \{i \in Y\}$$
$$\text{where}:$$
$$S^* = [S\ M\ (-M)]$$

D.

$$\text{maximize}: x_b$$
$$\text{subject to}:$$
$$\bar{a} \le S^* \le \bar{b}$$
$$l_i \le x_i \le u_i, \text{ for } i = 1, \ldots, n$$
$$x_i = 0, \text{ for } \{i > n | i \notin Y\}$$
$$0 \le x_i \le 10^3, \text{ for } \{i \in Y | i \ne j\}$$
$$x_j = 0$$
$$\text{where}:$$
$$S^* = [S\ M\ (-M)]$$

Figure S5: Flow chart for estimating minimum number of compounds needed to fix viability.

Growth-rate selection

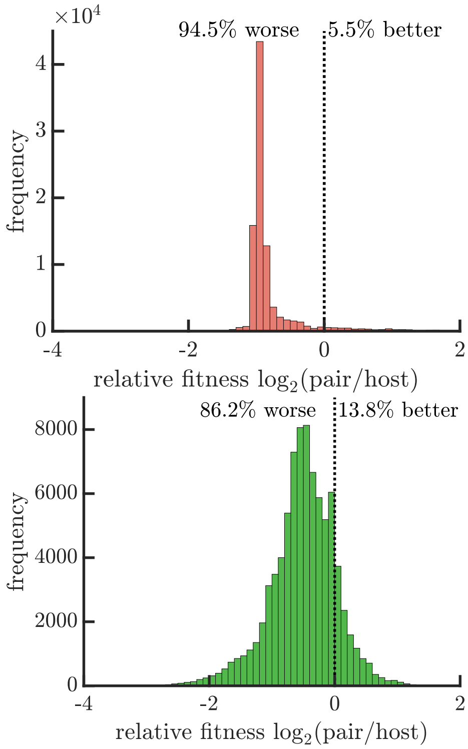

Figure S6: **Companion to Figure 5B.** The distributions are the same as in Figure 5B except the data is from KBase (top) or CarveMe (bottom). Both show that host-endosymbiont pairs more often have lower growth rates than their ancestral host metabolisms.

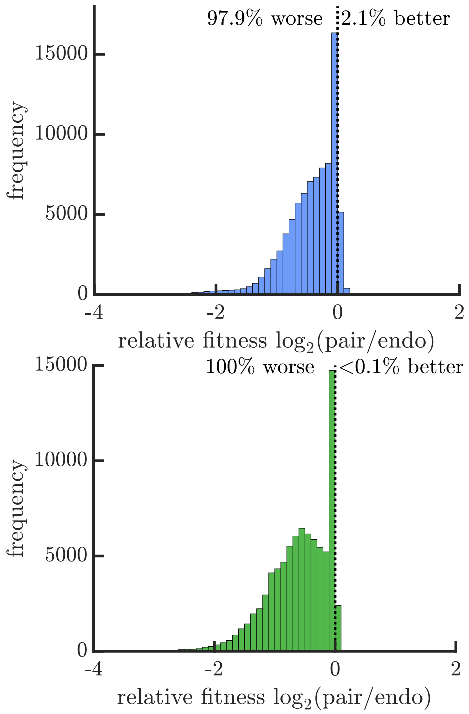

Figure S7: **Companion to Figure 5D.** The distributions are the same as in Figure 5D except the data is from AGORA (top) or CarveMe (bottom). Both show that host-endosymbiont pairs more often have lower growth rates than their ancestral endosymbiont metabolisms.

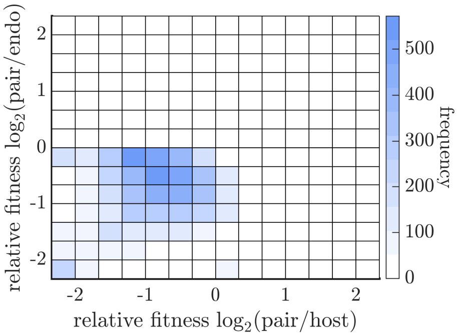

Figure S8: **Companion to Figure 5F.** The plot is the same as in Figure 4F except the data is from AGORA. We find a similar pattern in which the host-endosymbiont pair is less fit than both of its ancestral metabolisms.

Evolvability figures

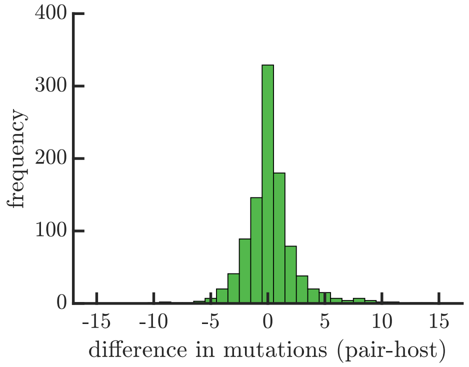

Figure S9: **Companion to Figure 6A.** The plot is the same as in Figure 6A except instead the data is from CarveMe instead of AGORA.

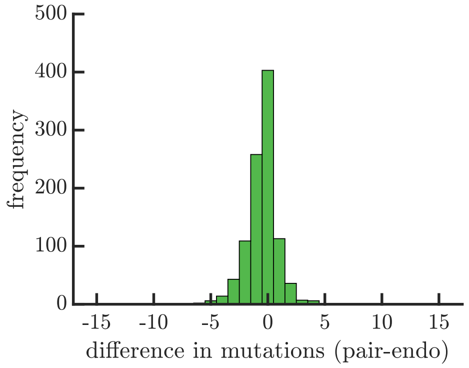

Figure S10: **Companion to Figure 6B.** The plot is the same as in Figure 6B except the data is from CarveMe instead of AGORA.

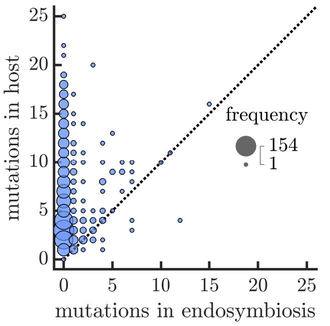

Figure S11: **Companion to Figure 6C.** The plot is the same as in Figure 6C except the data is from AGORA instead of CarveMe.

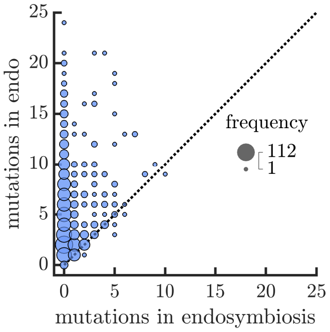

Figure S12: **Companion to Figure 6D.** The plot is the same as in Figure 6D except instead the data is from AGORA instead of CarveMe.

Archaea-bacteria pairings

The endosymbiosis underlying the evolutionary origins of eukaryotes likely took place between an archaea host and a bacteria endosymbiont. This motivated an exploration as to whether there is a difference in the viability of putative endosymbioses involving archaea-bacteria pairings compared to bacteria-bacteria pairings. We used taxonomical information from NCBI to assign prokaryotes as either archaea or bacteria. KBase was the only model collection in which we could find archaeal metabolic networks (139 models for archaea and 1498 models for bacteria). To evaluate whether pairing archaea with bacteria leads to different results in terms of viability, we considered all possible unique pairs of archaea with bacteria for a total of $208,222$ pairs. For each pair we assessed the viability of both possible configurations of endosymbioses, with archaea and bacteria swapping roles as host and endosymbiont (see Figure S13). When bacteria act as host the percentage of viable endosymbioses is similar to our findings in Figure 2, 65.7% vs 66.2% respectively. However, when archaea act as host the endosymbioses were less likely to be viable, 39.3%.

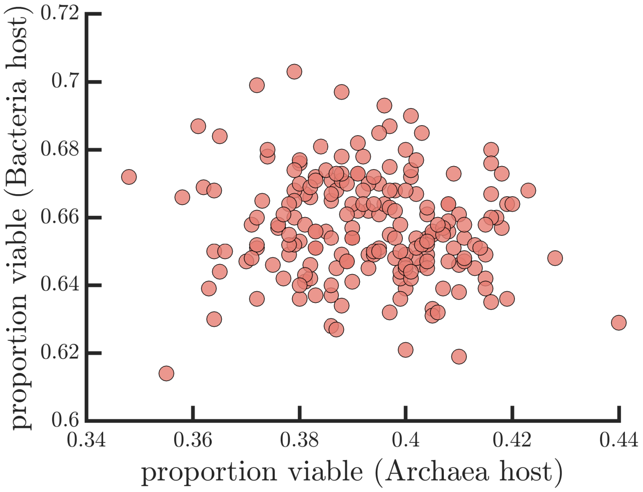

Figure S13: **Viability of endosymbioses in archaea-bacteria pairings.** Using the KBase collection we consider the viability of all possible endosymbioses formed by pairing archaea with bacteria ($208,222$ pairs). We grouped the pairs randomly into 208 sets of $\approx 1,000$ pairs. Each point represents a set and shows the proportion of endosymbioses that were viable when the archaea was the host (horizontal axis) versus when the bacteria was the host (vertical axis). For all sets endosymbioses with archaea hosts were less likely to be viable.

Since KBase was the only collection with archaea we cannot confirm that this is a general result. We did perform an additional analysis to identify a possible cause for the lower proportion of viable endosymbioses with archaea

host. We plotted the number of reactions in metabolic networks for archaea and bacteria in KBase and found that the distributions are different (see Figure S14). Archaea metabolic networks are often smaller than bacteria metabolic networks which could make them less likely to support another metabolism as an endosymbiont. Given the limited data on archaea metabolic networks it is not obvious whether their smaller sizes in KBase are actual or indicate an issue in methods for constructing archaea metabolic networks, e.g. they may be somehow incomplete.

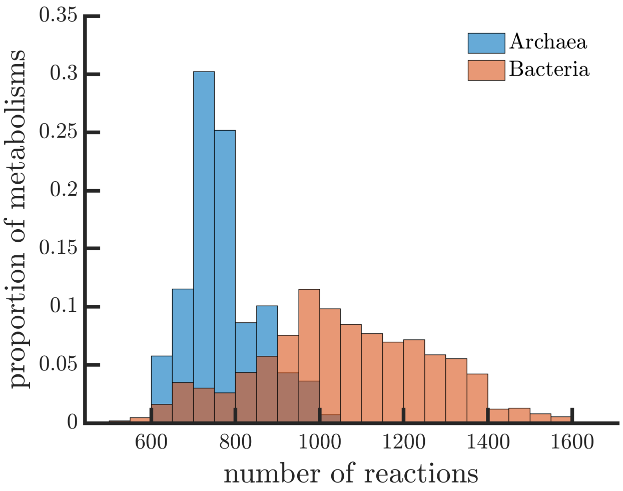

Figure S14: **Distributions of the number of reactions in archaea and bacteria metabolic networks.** Shown are the histograms for the number of reactions in metabolic networks from archaea (blue) and bacteria (red) in the KBase collection. We observe that the metabolic networks for archaea are often much smaller than bacteria which could impose a constraint on forming a viable endosymbiosis or represent a possible issue in archaea network construction.

Ecological interactions and viability

There are different possible ecological interactions between pairs of metabolisms, which could influence the viability of a putative endosymbiosis and its resulting growth rate. Here we chose to focus on two extremes: synergy and competition. In a synergy both metabolisms have a higher growth rate in each other's presence in the shared environment. Such scenarios may represent certain syntrophies that have been suggested to play a role in the evolution of mitochondria during eukaryogenesis. At the opposite extreme is competition in which both metabolisms experience a lower growth rate in each other's presence in the shared environment. We searched each collection for cases of competition and syntrophy and report the results of our analyses below.

CarveMe

Using CarveMe models we found no examples of syntrophy in our original dataset of paired metabolisms. We searched an additional 200,000 random pairs of metabolisms and found only one case of syntrophy. We evaluated the viability of an endosymbiosis formed from these syntrophic metabolisms and found it was viable in only one configuration, with a growth rate smaller than the ancestors growing together. We note that the limited observations of syntrophy in CarveMe models likely reflect the way in which default environments were selected rather than issues with the metabolic models themselves.

KBase

In KBase models we could not unambiguously identify examples of syntrophy and competition because of the same issues that interfered with our analyses in Figure 5, i.e. models from KBase often yield multiple possible growth rates for two metabolisms growing together in a shared environment.

AGORA

For the remaining collection, AGORA, we searched another 200,000 random pairs of metabolisms to find instances of syntrophy. We found $4,082$ examples of syntrophies where each member of a pair of metabolisms have an increased growth rate when grown together. As a comparison we also found $10,011$ examples of competition where each member of a pair of metabolisms have a decreased growth rate when grown together. We compared the proportion of endosymbioses that are viable when the ancestral metabolisms are syntrophic versus competitive. We found that viability was lower in syntrophic pairs (46.47%) versus competitive pairs (61.38%). We also found that the original type of ecology (syntrophy versus competition) did not affect the resulting fitness of a viable endosymbiosis compared to its ancestors. In both cases the endosymbiosis was less fit than both its ancestors in $82-86\%$ of cases, similar with our original findings in Figure 5E of $\approx 88\%$. And in both cases of syntrophy and competition

it was rare to find an example where an endosymbiosis had a higher growth rate than both of its ancestors (0 observations in syntrophy and 1 in competition).

Interpretation of the results

We lack sufficient data in KBase and CarveMe to confirm the generality of our observation from AGORA models that syntrophic metabolisms are less likely to produce viable endosymbioses. However, there may be a reason that this result could be more general. If we consider the case of two identical metabolic networks growing in the same environment then we would expect them to compete because they require the same resources. But since they both need the same resources if they formed an endosymbiosis then the host would be guaranteed to transport all of the compounds needed for the endosymbiont (because they are the same metabolically), thereby preventing a common cause of nonviability. Indeed our analyses from the Supplementary section "Predicting viability" confirms that metabolisms paired with themselves frequently result in viable endosymbioses. If instead we consider a syntrophy then the two metabolic networks should not be limited by the same resource, otherwise they would compete for that resource. In addition, they must also have different pathways to provide a growth benefit to each other. These metabolic differences create an opportunity for a mismatch between what compounds the host can transport inside of its cell and those the endosymbiont requires. This reasoning suggests that syntrophic interactions may make an endosymbiosis more difficult—it is important to note that the reduction in viability is relatively small and unlikely to account for the rarity of prokaryotic endosymbioses.

These analyses raise a question concerning the diversity of organisms present in the collections. If very similar organisms are represented in model collections or if similar strains of the same organism appear often in a collection then this could bias the results— if most metabolisms are from the same species then competitive interactions would dominate. We looked through the model collections at the organisms represented and found that while different strains of the same species do appear in collections, it mostly occurs for a few well-studied species, e.g. *E. coli*. The vast majority of metabolic models correspond to species that only occur once within a collection: 72.4% of models in AGORA, 89.1% of models in KBase, and 94.4% of models in CarveMe. The fact that models in CarveMe and AGORA (the opposite ends of this spectrum) consistently return similar predictions suggests that our findings are unlikely to be driven by biases associated with double counting of metabolisms. Nevertheless, we performed an additional analysis to evaluate whether issues of double counting may bias our results. We went through each model collection and identified species that were unique to that collection, that is, those species that were only found in a single collection and occurred only once (a single strain was present). We then used this set of unique networks to evaluate the viability of 10,000 random pairings (note that enough networks were present in each collection that more than 10,000 unique pairings were possible). In all three collections we found similar levels of viability as in our original study: 63.5% in CarveMe, 59.5% in KBase,

and 56.7% in AGORA, which further supports the idea that double counting did not significantly bias our analyses.

References

[1] Stefanıa Magnúsdóttir et al. "Generation of genome-scale metabolic reconstructions for 773 members of the human gut microbiota". In: *Nature biotechnology* 35.1 (2017), pp. 81–89.

[2] Adam P Arkin et al. "KBase: the United States department of energy systems biology knowledgebase". In: *Nature biotechnology* 36.7 (2018), pp. 566–569.

[3] Daniel Machado et al. "Fast automated reconstruction of genome-scale metabolic models for microbial species and communities". In: *Nucleic acids research* 46.15 (2018), pp. 7542–7553.
